# LegumeSSRdb: A Comprehensive Microsatellite Marker Database of Legumes for Germplasm Characterization and Crop Improvement

**DOI:** 10.3390/ijms222111350

**Published:** 2021-10-21

**Authors:** Naveen Duhan, Rakesh Kaundal

**Affiliations:** 1 Department of Plants, Soils and Climate, CAAS, Utah State University, Logan, UT 84321, USA; naveen.duhan@usu.edu; 2 Center for Integrated BioSystems (CIB), CAAS, Utah State University, Logan, UT 84321, USA; 3 Department of Computer Science, CoS, Utah State University, Logan, UT 84321, USA

**Keywords:** Fabaceae, genetic linkage map, genomics, *in silico* data mining, marker-assisted selection, plant breeding and genetics, SSR

## Abstract

Microsatellites, or simple sequence repeats (SSRs), are polymorphic loci that play a major role as molecular markers for genome analysis and plant breeding. The legume SSR database is a webserver which contains simple sequence repeats (SSRs) from genomes of 13 legume species. A total of 3,706,276 SSRs are present in the database, 698,509 of which are genic SSRs, and 3,007,772 are non-genic. This webserver is an integrated tool to perform end-to-end marker selection right from generating SSRs to designing and validating primers, visualizing the results and blasting the genomic sequences at one place without juggling between several resources. The user-friendly web interface allows users to browse SSRs based on the genomic region, chromosome, motif type, repeat motif sequence, frequency of motif, and advanced searches allow users to search based on chromosome location range and length of SSR. Users can give their desired flanking region around repeat and obtain the sequence, they can explore the genes in which the SSRs are present or the genes between which the SSRs are bound design custom primers, and perform *in silico* validation using PCR. An SSR prediction pipeline is implemented where the user can submit their genomic sequence to generate SSRs. This webserver will be frequently updated with more species, in time. We believe that *legumeSSRdb* would be a useful resource for marker-assisted selection and mapping quantitative trait loci (QTLs) to practice genomic selection and improve crop health. The database can be freely accessed at http://bioinfo.usu.edu/legumeSSRdb/.

## 1. Introduction

Legumes are the second most essential group of crops with around ~800 genera and ~20,000 known legume species in the world. They are among the few species which can convert nitrogen available in the air to plant-usable form and are capable of increasing the nitrogen content in the soil which, in turn, helps in nitrogen fertilization for other crops [1]. Legumes play a significant role in natural ecosystems, agriculture, and agroforestry. They play an important role in both animal and human food, providing approximately one-third of human nitrogen. An important nutritional aspect of legumes is the high concentration of protein, oil, and starch in their seeds, and they play a significant role in animal forages, and livestock as well as human consumption. *Medicago truncatula* and *Lotus japonicas* are two major model organisms that have been used to elucidate the genetic basis of legume-rhizobial symbiosis, which is used to fix atmospheric nitrogen for plant use, whereas legumes such as *Phaseolus vulgaris* and *Glycine max* are major staple crops in various parts of the world.

With the increase in water-stressed areas in the world, drought problems are likely to increase in legume species [2]. It is important to study legumes and come up with solutions to cultivate more drought-tolerant legumes. Thus, it is important to understand the fundamental mechanisms of these species under different biotic and abiotic stresses. The advancement in molecular marker technology and next-generation sequencing technologies has increased the scope for crop improvement as it has become relatively easier for researchers to study any given species of interest on a genome-scale. 

In conventional plant breeding, the genetic selection of plants is decided by the parents and influenced by different environmental conditions [3]. In traditional plant breeding, alleles are mixed over generations and new combinations are produced, which contribute to the selection process to achieve higher quality. Marker-assisted selection (MAS) has become common in many crop breeding programs in recent years [4,5,6,7]. The discovery of a DNA-based marker that is closely linked to the target trait is a prerequisite for using MAS. In many crops, the marker method of choice for quantitative trait loci (QTL) mapping has long been microsatellite markers (SSRs). Microsatellites are tandem repeats of 1–6-nucleotide-long DNA units that are flanked by identical genome sequences but occur more frequently in the non-genic region [8]. Per generation, the mutation rate of SSRs ranges between 10^−3^ and 10^−6^ [9], which increases with the length of the repeat unit [10]. They are extremely flexible, low-cost, highly insightful molecular markers, and based on PCR, consistent with high-frequency polymorphism [11]. These are one of the markers among various genetic markers such as RFLP (restriction fragment length polymorphism), RAPD (random amplification of polymorphic DNA), AFLP (amplified fragment length polymorphism), and SNP (single nucleotide polymorphism, used for germplasm characterization). SSRs have many applications such as genetic diversity assessment, gene mapping, marker-assisted selection [12], the study of population and phylogenetic relationships and bio-invasions, and disease control [11]. 

SSR screening using genomic libraries are time-consuming and expensive on a large scale; in recent years, the *in silico* approach of marker development has paved a path for researchers to make the SSR development viable and fast [13]. Therefore, a user-friendly database of all available genomic data of the legume species can be a valuable genomic resource for legume cultivation improvement and characterization, and bearing in mind the importance of SSRs for crop improvement in legumes. Here, we present *legumeSSRdb*, a comprehensive web resource integrated with a wide range of services such as SSR prediction, primer design for genotyping along with ePCR-based polymorphism discovery, JBrowse visualization, and many other enriching features. 

## 2. Results

### 2.1. Cross-Species Comparison of Legume Species SSRs

A total of 3,706,276 microsatellites were predicted from 13 legume species for the development of the *legumeSSRdb* web-resource. The highest numbers of SSRs were predicted in *Arachis hypogaea*, whereas *Trifolium pratense* had the fewest SSRs (Table 1). The number of SSRs is strongly associated with the size of the genome; the larger the genome, the greater number of SSRs are predicted, except for the atypical case of *Phaseolus vulgaris.* Generally, species with large genome sizes tend to have low SSR frequency (SSRs/MB) [14]. However, there was no link found between the SSR density and genome size among the SSRs discovered in our analysis of 13 legume species. This is consistent with some of the recent findings that found no relation between the genome size and SSR density, and that the genome size differences may influence the degree of microsatellite repeats in the genome [15,16,17,18].

### 2.2. Characterization of the Perfect SSRs

SSRs repeating ≥ 15 times were termed as perfect SSRs. On average, around 30–40% of SSRs found were perfect. Out of these perfect SSRs, 25% were located in the coding regions of the genome and 75% were present in non-coding regions. Among the 13 species, *Phaseolus vulgaris* (66%) had the highest and *Vigna unguiculata* had the lowest percentage of perfect SSRs. *Trifolium pratense* has the highest percentage of perfect SSRs present in coding regions of the genome and *Cicer arietinum* has the highest percentage of perfect SSRs present in non-coding regions (Table 1).

### 2.3. Characterization of SSRs by Motif Type

All the predicted SSR loci were categorized into six categories: monomers, dimers, trimers, tetramers, pentamers, and hexamers. Among all the species, around 85% of SSRs comprised monomers and dimers. *Medicago truncatula* had the highest percentage of monomeric repeat, whereas *Lupinus albus* had the lowest percentage of monomeric repeat; *Lupinus albus, Lupinus angustifolius,* and *Phaseolus vulgaris* had more dimeric repeat than monomeric repeat (Table 2, Supplementary Figure S1). There has been a high abundance of monomeric repeats in almost all genomes, which may be due to the inherent limitations of next-generation sequencing (NGS) methods used for data generation [17]. Likewise, dimeric repeat also recorded a higher abundance in other crops [19,20]. For trimeric repeat, *Lupinus angustifolius* had the highest percentage and *Medicago truncatula* had the lowest percentage. In the case of tetrameric repeat, *Trifolium pretense* had the highest percentage and *Medicago truncatula* had the lowest percentage. *Lupinus albus* had the highest percentage (16.4%) of pentameric repeat, whereas *Medicago truncatula* and *Vigna unguiculata* had the lowest percentage (0.1%). For hexameric repeat, *Lupinus angustifolius* had the highest percentage (9.5%) followed by *Lupinus albus* and *Cicer arietinum* (0.3%), whereas *Glycine max*, *Medicago tranctula*, *Trifolium pratense*, *Phaseolus vulagaris*, and *Vigna anguaris* has the lowest percentage (0.1%). In all SSR classes, it was observed that longer repeats were less abundant; a decreased trend in SSR frequency with an increased trend in their repeat number has been observed in other species [18,21].

### 2.4. Functional Annotations of Predicted SSRs

The predicted SSRs in each species were mapped to their corresponding annotation file to classify genic and non-genic SSRs. Furthermore, genic SSRs were classified into exons, 5′ UTR and 3′ UTR regions. For non-genic SSRs, their closest genes were also assigned. Around 20–25% of the genic SSRs were present in each species (Supplementary Figure S2). *Trifolium pratense* had the highest percentage (36%) of SSRs present in the genic region, whereas *Cicer arietinum* had the lowest percentage (13%) of genic SSRs.

### 2.5. Web Genomic Resource: legumeSSRdb

**Figure 1:**
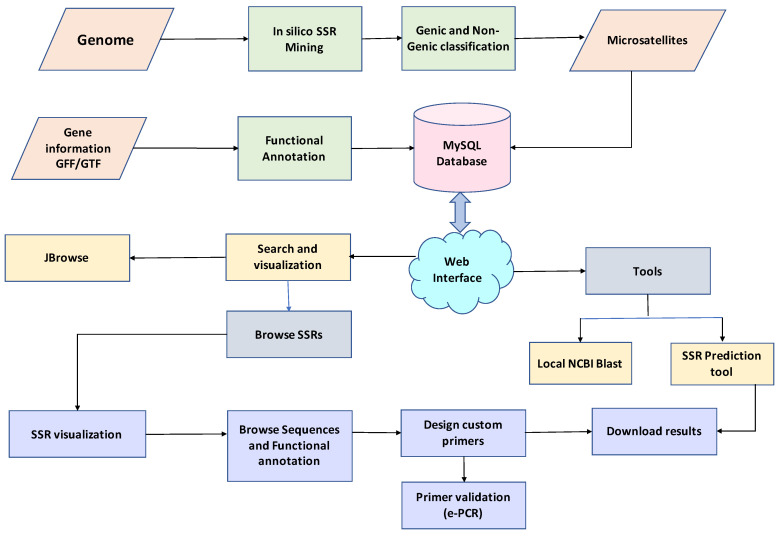
Overall workflow of *legumeSSRdb*.

The legume SSR web genomic resource (*legumeSSRdb*) was developed using three-tier architecture. This is the first comprehensive legume SSR resource containing 13 species. The web resource comprises seven tabs: Home, About, Species, Tools, JBrowse, Help, and Contact. Predicted SSRs can be searched based on the region in the genome, chromosome number, motif type, and repeat motif sequence. In the advanced query, a user can choose more parameters such as the range of the number of repeat units (how many times a motif should repeat), range of length of the SSR, and chromosome location range (the specific location of the genome with a start and an end). The result page displays real-time visualization of the searched result with graphs and tables. The result table can be sorted based on motif start, motif end, size, motif type, gene location, etc. Users can download results in a text file with a download result button, the flanking region of the selected SSRs can be viewed with the ‘get sequence’ option, and the ‘design primer’ button can be used to design primers for selected SSRs. The primer page displays designed primers in the flanking region and results can be downloaded in text format. The designed primers can be searched for cross-species transferability using an e-PCR option. The tool tab provides three tools: SSR prediction, BLAST, and JBrowse. The tool *miSATminer* has been implemented with custom scripts to design the SSR markers for user input sequences, whereas users on the BLAST search page can execute similarity searches. The 13 legume species genome sequences can be viewed with gene and SSR coordinates on the chromosome using the JBrowse tool option. The Help tab contains a tutorial for using the database efficiently and frequently asked questions. A workflow of searching *legumeSSRdb* is illustrated in (Figure 1).

## 3. Discussion

*LegumeSSRdb* is the first comprehensive database of legumes represented by 13 species containing 3,706,276 *in silico* predicted SSR markers. This study clearly shows that SSR mining can be performed more effectively via the computational approach [22]. These markers are ubiquitously spread across the whole chromosome sets; therefore, they may be a better example in the form of a molecular marker for the study of variability analysis. This strategy has the advantage of location specificity over chromosome, and it can be used as a specific gene molecular marker [23]. Designed SSR primers may be used to identify the QTL/candidate gene, generate a linkage map, hybrid development, and characterization of germplasm. Many studies have been reported on the use of microsatellite markers for mapping various quantitative traits in plants [24,25,26,27,28]. The SSR markers present in the flanking region of the genes can be used for marker-assisted selection (MAS) and germplasm improvement [29]. SSR markers have been used in the characterization of genotypes for early leaf spot (ELS) resistance, yellow mosaic virus resistance genes, and to identify QTLs for flowering traits and other traits of interest [30,31]. Varieties with identical morphological features are highly difficult to discern from the phenotypic study. Previous research has used SSRs to address these difficulties for variety characterization, linkage mapping, trait improvement, molecular breeding, hybrid cultivar development, improved variety development phylogenetic, and taxonomic comparisons [32,33]. Several whole genomes of numerous plant species are available in the public domain and offer the potential to research the cross-species transferability in closely related plants; it can aid in the cloning of candidate genes from different species and orthologous loci within different species.

Since SSRs have many significant applications, a need exists for a full-fledged database resource of microsatellite marker development for legume species. Although some web resources provide information related to legumes, they lack microsatellite markers, SSR prediction, e-PCR primer design, visualization, and other related information. For example, in PMDBase, there are 15 species of legumes present with marker data, but this tool lacks advanced microsatellite search criteria; however, *legumeSSRdb* provides advanced search criteria such as frequency, motif type, repeat type, chromosome range, etc. Other than *legumeSSRdb*, no database provides a tool for checking the cross-species transferability of markers. As shown, our database could serve as a complementary resource to further advance plant genomics and breeding research in the legume species. The comparison of *legumeSSRdb* with similar existing tools is illustrated in Table 3.

## 4. Materials and Methods

### 4.1. Data Collection

At first, the whole genome sequences of 13 legume species with annotations were downloaded. Out of these, 6 species (*Glycine max, Cicer arietinum, Medicago truncatula, Trifolium pratense, Phaseolus vulgaris, Vigna unguiculata*) were downloaded from Phytozome (https://phytozome.jgi.doe.gov/pz/portal.html, accessed on 11 December 2020) whereas 7 species (*Arachis hypogaea, Arachis ipaensis, Cajanus cajun, Lupinus albus, Lupinus angustifolius, Vigna angularis, Vigna radiata*) were downloaded from NCBI (https://www.ncbi.nlm.nih.gov/, accessed on 5 January 2021). 

### 4.2. In Silico Simple Sequence Repeat Mining and Functional Annotation

All the 13 species genome sequences were processed with *miSATminer*, our in-house developed Perl script for SSR prediction [34]. Microsatellites were identified with parameters such as 10 repeat units for mono, and 5 repeat units for the di, tri, tetra, penta, and hexa. Predicted SSRs were classified in the genic region and non-genic regions and further annotated using gene annotation files. 

### 4.3. Webserver Development and Web Interface

Legume SSR database (*legumeSSRdb*) is a three tier-based relational database webserver with a client-tier, middle-tier, and database-tier. Predicted SSRs from 13 species were stored in a database using MySQL and accessed using PHP and Apache. A user-friendly web interface was developed using HTML5, Bootstrap4 CSS, JavaScript, and Jquery. Realtime visualization of data was implemented using several JS chart libraries, such as ChartJS, Morris, Flot, C3 charts, etc. Primer3 was implemented for real-time primer-designing. Genomic sequence and annotation visualization was implemented through JBrowse. For the SSR prediction to a user-given query, the *miSATminer* script was implemented in the backend. NCBI local BLAST and e-PCR was also implemented for similarity search and cross-species transferability, respectively. The overall workflow of the web resource is presented in Figure 1.

## 5. Conclusions

*LegumeSSRdb* is the first SSR database that has been designed with cutting edge GUI features and integrates all the services required for performing end-to-end marker selection in legume species. *LegumeSSRdb* contains 3,706,276 putative microsatellites from 13 legume species. These markers can be used with cross-species transferability to cater to the need for molecular markers for legume species where the whole-genome sequence data are not available. This genomic web resource can be of great value to the global community. The webserver facilitates the ability to search, predict, analyze, and visualize the SSRs of 13 legume species. Features exists such as the ability to create custom primers based on user-defined amplicon size and *in silico* validation, the real-time graphical visualization of SSR results, exploring SSR sequences by adjusting the flanking region, and identifying related genes and exploring their functional annotation information which is unique to *legumeSSRdb*. This web server also allows users to submit a genomic sequence of their interest to predict SSRs by adjusting parameters and design primers. With high-performance cluster computing on the backend, *legumeSSRdb* provides quick and flawless performance. This can be used for chromosome-wise microsatellite locus mining and primer designing for genic and non-genic FDM-SSRs for rapid genotyping (FDM, functional domain markers). This can also be used to facilitate the detection of polymorphisms by e-PCR that is most economically needed in future re-sequencing ventures. This web resource is not limited to knowledge discovery research such as genetic linkage mapping, QTL identification, etc., but can also be used for marker-assisted breeding and germplasm improvement of legume species. This web resource will be extended to more legume species in the future. The webserver is freely available at (http://bioinfo.usu.edu/legumeSSRdb/).

## Figures and Tables

**Table 1:** Statistics of species-wise microsatellites based on genome size, number of base-pairs, number of SSRs per mega-base-pairs, genomic location, and the number of repeat units.

Genome	Size MB	No. of Base Pairs	No. of SSRs	Freq/Mbp	Perfect SSRs (Repeat Units ≥ 15)						
					Count	%	Freq/Mbp	Genic	%	Non-Genic	%
*Glycine max*	974	973,419,153	475,123	488.1	150,682	31.7	154.8	35,588	23.6	115,094	76.4
*Cicer arietinum*	350	350,719,855	193,672	552.2	51,797	26.7	147.7	8014	15.5	43,783	84.5
*Medicago truncatula*	391	390,874,780	238,882	611.1	68,657	28.7	175.6	18,109	26.4	50,548	73.6
*Trifolium pratense*	192	192,330,821	105286	547.4	23,674	22.5	123.1	10,993	46.4	12,681	53.6
*Phaseolus vulgaris*	520	520,399,038	193,735	372.3	127,463	65.8	244.9	16,083	12.6	111,380	87.4
*Vigna unguiculata*	481	481,347,227	290,479	603.5	54,679	18.8	113.6	9510	17.4	45,169	82.6
*Arachis hypogaea*	2600	2,570,012,282	1,009,984	393.0	319,463	31.6	124.3	50,863	15.9	268,600	84.1
*Arachis ipaensis*	1400	1,359,188,642	437,350	321.8	99,538	22.8	73.2	21,942	22.0	77,596	78.0
*Cajanus cajun*	250	250,588,641	165,919	662.1	56,287	33.9	224.6	12,504	22.2	43,783	77.8
*Lupinus albus*	480	480,287,150	146,505	305.0	62,895	42.9	131.0	13,542	21.5	49,353	78.5
*Lupinus angustifolius*	476	476,300,322	132,282	277.7	54,187	41.0	113.8	12,508	23.1	41,679	76.9
*Vigna angularis*	377	377,395,406	140,751	373.0	38,517	27.4	102.1	8514	22.1	30,003	77.9
*Vigna radiata*	338	337,474,823	176,308	522.4	61,388	34.8	181.9	12,866	21.0	48,522	79.0

**Table 2:** Distribution of SSRs based on motif type. Where ‘All’ represents the percentage of all motifs in the genome, whereas ‘P’ represents the perfect motif-type percentage.

Genome	Mono%		Di%		Tri%		Tetra%		Penta%		Hexa%	
	All	P	All	P	All	P	All	P	All	P	All	P
*Glycine max*	51.1	15.9	39.3	53.8	8.6	27.3	0.8	2.5	0.2	0.5	0.1	0.2
*Cicer arietinum*	53.3	15.1	31.7	38.1	12.6	40.3	1.6	5.2	0.4	1.2	0.3	1.1
*Medicago truncatula*	67.6	41.8	25.5	34.1	6.1	21.4	0.6	2.3	0.1	0.4	0.1	0.2
*Trifolium pratense*	62.7	13	25.5	34.7	9.7	43.1	1.8	8.2	0.2	1.1	0.1	0.4
*Vigna unguiculata*	49.4	8.4	39.3	52.0	10.3	36.7	0.7	2.5	0.1	0.5	0.2	0.6
*Phaseolus vulgaris*	40.2	5.5	46.6	64.7	11.6	26.4	0.9	2.2	0.6	1.3	0.1	0.3
*Arachis hypogaea*	44.3	11.9	40.5	40.2	13.2	42.1	1.4	4.5	0.4	1.4	0.2	0.6
*Arachis ipaensis*	47.4	9.9	39.2	31.7	11.1	49.2	1.6	6.9	0.5	2.3	0.2	0.8
*Cajanus cajun*	46.3	17.5	44.5	56.0	7.7	22.7	1.1	3.3	0.2	0.5	0.2	0.7
*Lupinus albus*	25.0	5.3	49.4	35.4	8.0	18.7	1.0	2.3	16.4	38.3	0.3	0.6
*Lupinus angustifolius*	25.9	2.3	49.3	49.0	14.0	44.4	0.9	2.9	0.4	1.4	9.5	30.2
*Vigna angularis*	46.5	5.9	43.1	56.4	9.3	34.0	0.7	2.5	0.3	1.1	0.1	0.5
*Vigna radiata*	52.4	12.6	38.6	61.7	7.8	22.6	0.8	2.3	0.3	0.8	0.1	0.2

**Table 3:** Comparison of *legumeSSRdb* with other related databases.

Features	legumeSSRdb	CicArMiSatDB	Legumeinfo	LegumeIP	PMDbase
Number of species	13	1	22	21	15
Microsatellites	Yes	Yes	No	No	Yes
Microsatellite Search Criteria	Yes (Advanced)	Limited	No	No	Limited
Microsatellites results—Graphical visualization	Yes	No	No	No	No
Genic and non-genic classification of SSRs	Yes	No	No	No	No
Primer Designing	Yes (Custom)	Yes (Predesigned)	No	No	Yes (Predesigned)
Primer Validation using e-PCR	Yes	No	No	No	No
BLAST	Yes	Yes	Yes	Yes	Yes
Blast result graphical visualization	Yes	No	No	No	No
Genome Browse	Yes	Yes	No	No	Yes
SSR Predictor	Yes	No	No	No	Yes
Primer Designing for predicted SSRs	Yes	No	No	No	No
Functional Annotation	Yes	No	Yes	Yes (In-depth)	No

## Data Availability

All the data is available at http://bioinfo.usu.edu/legumeSSRdb/.

## Supplementary Materials

The following are available online at https://www.mdpi.com/article/10.3390/ijms222111350/s1.
