# Supplementary material for: LegumeSSRdb: A Comprehensive Microsatellite Marker Database of Legumes for Germplasm Characterization and Crop Improvement
Source: Int J Mol Sci. 2021 Oct 21;22(21):11350. doi: 10.3390/ijms222111350 (PMC8583334; doi:10.3390/ijms222111350)
Supplement: Supplementary file 1 [file ijms-22-11350-s001.zip › ijms-1347473-supplementary.pdf]

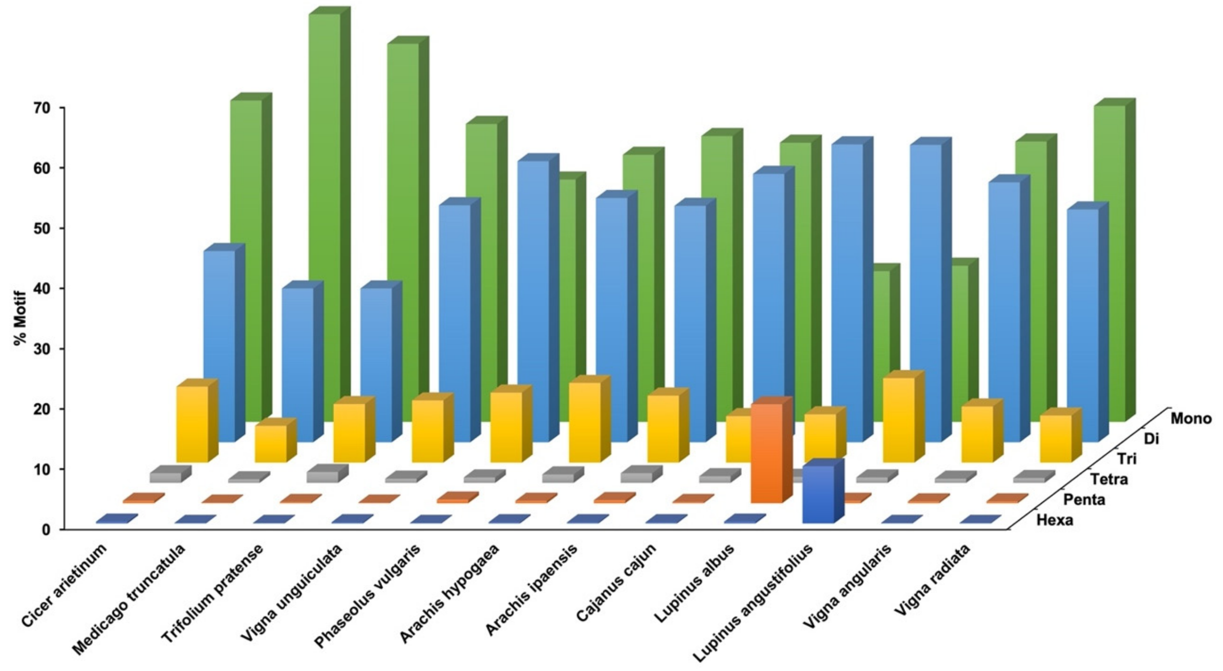

Supplementary Figure S1*:* Line chart to compare the distribution of SSRs in different motif types between 13 legume species.

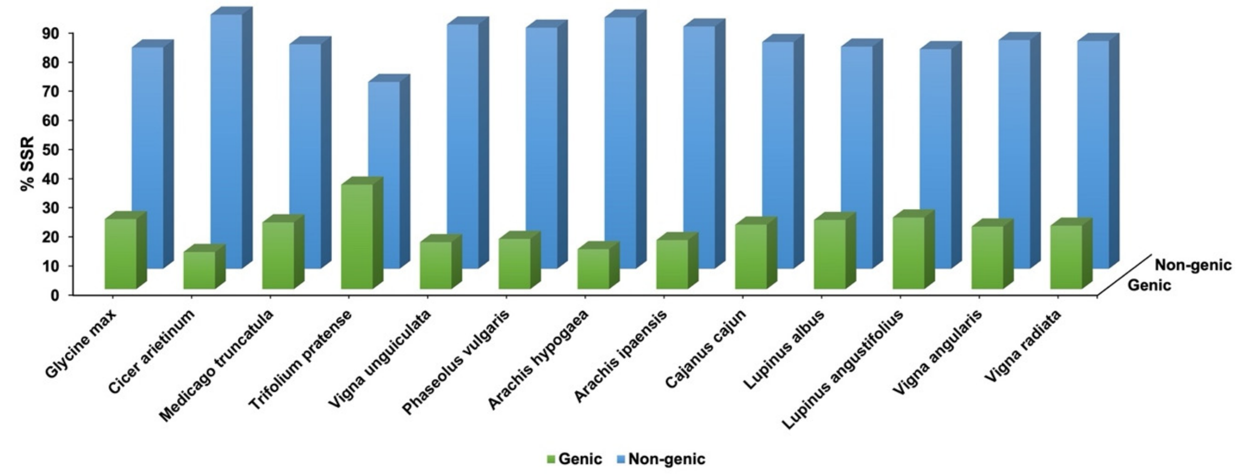

Supplementary Figure S2: Distribution percentage of SSRs in genic and non-Genic regions of their genomes.
